# Pharmacokinetic study of a novel oral formulation of S-adenosylmethionine (MSI-195) in healthy subjects: dose escalation, food effect and comparison to a commercial nutritional supplement product

**DOI:** 10.1186/s40360-020-00466-7

**Published:** 2020-12-14

**Authors:** Beth R. Cameron, Ludvina Ferreira, I. David MacDonald

**Affiliations:** MSI Methylation Sciences Inc, Suite 300, 15300 Croydon Drive, Surrey, BC V3Z 0Z5 Canada

**Keywords:** Pharmacokinetics, Ademetionine, Adomet, S-adenosylmethionine, Formulation, Relative bioavailability

## Abstract

**Background:**

A novel, high bioavailability oral, enteric coated tablet formulation of S-adenosylmethionine (MSI-195) has been developed for life science application. The present research reports on a Phase 1 study to (i) determine the safety of single doses of MSI-195 (ii) to determine the dose proportionality of MSI-195 at doses of 400, 800 and 1600 mg (iii) determine the pharmacokinetics of MSI-195 compared with a commercial reference product (SAM-e Complete™) over 24 h and (iv) to determine the effect of food on the pharmacokinetic profile of MSI-195 in human subjects.

**Methods:**

This study was a pharmacokinetic and safety evaluation of MSI-195 and a commercial comparator broken into two stages. The first stage was an exploratory single ascending dose design of MSI-195 in 8 healthy normal male volunteers. The second stage was a single dose evaluation, targeting 26 male and female volunteers at set doses of MSI-195 and commercial comparator in a cross-over design followed by a food effect study on MSI-195. Plasma samples were collected and assayed for S-adenosylmethionine using a validated HPLC method with MS/MS detection. The main absorption and disposition parameters were calculated using a non-compartmental approach with a log-linear terminal phase assumption. Statistical analysis was based on an ANOVA model or t test as appropriate.

**Results:**

MSI-195 was found to be generally well tolerated with an adverse event profile similar to the SAM-e Complete™ comparator product. The relative bioavailability of MSI-195 was approximately 2.8-fold higher than SAM-e Complete based on area under the curve (AUC) ratios for the two products and the MSI-195 formulation exposure based on AUC was found to be approximately dose proportional. There was a significant food effect for MSI-195 with a delayed time to maximum absorption T_max_, going from 4.5 h under fasted conditions to 13 h under fed conditions, and area under the curve with food reduced to 55% of that seen under fasting conditions.

**Conclusions:**

The overall conclusion was that MSI-195 was well tolerated and has markedly higher bioavailability compared with both the SAM-e Complete™ commercial product tested and, on a per mg basis, products reported in other literature.

**Trial registration:**

ClinicalTrials.gov, identifier NCT04623034. Retrospectively registered Nov 9, 2020.

## Background

S-adenosylmethionine (SAMe, generic name Adomet) is a naturally occurring, physiologically active molecule that is distributed in all body tissues. SAMe has been studied pharmacologically, with varying levels of clinical evidence supporting efficacy, in numerous indications including depression, liver disease and osteoarthritis [1–4]. More recently, the molecule is being studied pre-clinically as a potential therapeutic in oncology [5–8].

MSI-195 is a proprietary, oral formulation of SAMe that has been researched, developed and patented by Methylation Sciences. This formulation was developed and has been studied in the United States under an FDA IND in a multicenter, randomized, double-blind, placebo-controlled Phase 2 depression clinical trial (ClinicalTrials.gov NCT01912196). [9, 10]

The history of S-adenosylmethionine pharmacology began in the 1970′s when it was studied as a parenteral product in Europe, and subsequently as an oral formulation [11]. More recently, oral formulations of SAMe have been commercialized in the dietary/nutritional supplement fields. Current oral formulations are enteric coated, which helps with tolerability and bioavailability however, even with that, oral bioavailability is low. Yang and co-workers determined an AUC_0-24_ Oral/IV ratio was determined to be 2.60% and 2.14% in men and women respectively [12].

Oral dosing of S-adenosylmethionine in clinical studies for depression is most frequently 1600 mg/day [13]. (dosing is calculated based on the amount of the free base). To deliver that dose, 4 large tablets of approximately 1 g each are required. This is a significant pill burden and also comes at a significant expense.

The objectives of this clinical study were to (i) determine the safety of single doses of MSI-195 (ii) to determine the dose proportionality of MSI-195 at doses of 400, 800 and 1600 mg (iii) determine the pharmacokinetics (PK) of MSI-195 compared with a commercial reference product (SAM-e Complete™ and (iv) to determine the effect of food on the pharmacokinetic profile of MSI-195 in human subjects.

## Methods

### MSI-195 formulation

The MSI-195 formulation is a novel patented [14, 15] tablet that is comprised of a core tablet, a seal coat and an enteric coat. The core contains S-adenosylmethionine Disulphate Tosylate (OmniaBios), microcrystalline cellulose, sodium starch glycolate, colloidal silicon dioxide, magnesium stearate and the GRAS excipient propyl gallate. The seal coat consists of Opadry AMB and ferric oxide and the enteric coating is Eudragit L30D55(Evonik). MSI discovered that formulations of SAM-e including propyl gallate have enhanced systemic absorption [14]. The test article used in the present study has a dosage strength of 400 mg (based on the weight of the free base). The SAM-e counter-ions are disulfate tosylate and the overall tablet weight is approximately 1.2 g.

### SAM-e complete™ commercial comparator

The SAM-e Complete™ commercial comparator is manufactured by Pharmavite under the NatureMade™ brand. The dosage strength is 400 mg (based on the free base). Total tablet weight is approximately 1 g. Test material was purchased in the United States at Costco.

### Study design

This study was a pharmacokinetics and safety evaluation of the MSI-195 proprietary S-adenosylmethionine oral dosage form and a commercial comparator consisting of two stages. (see Table 1) The first stage was an exploratory single ascending dose design of MSI-195 in 8 healthy normal male volunteers. The second stage was a more comprehensive single dose evaluation, targeting 26 male and female volunteers at set doses of MSI-195 and commercial comparator in a cross-over design followed by a food effect study on MSI-195. Further design details are listed below.

**Table 1:** Description of the test articles and dosing for the 2 stages of this pharmacokinetic study

S-adenosylmethionine Test Article Name	Dosage Form/ Route of Administration	Stage 1 dosing	Stage 2 dosing
MSI-195^a^	Tablet/ Oral	• 400 mg (1 x 400 mg)	• 800 mg (2 x 400 mg)
		• 800 mg (2 x 400 mg)	
		• 1600 mg (4 x 400 mg)	
SAM-e Complete^TMb^	Tablet/Oral	n/a	• 1600 mg (4 x 400 mg)

^a^MSI-195 is Methylation Sciences’ proprietary oral enteric coated tablet formulation of S-adenosylmethionine

^b^SAM-e Complete™ is a commercial dietary supplement oral enteric coated tablet formulation manufactured by Pharmavite in the United States under the NatureMade™ brand

#### Stage 1 design

In each period of this stage of the study, ascending single doses of 400 mg, 800 mg and 1600 mg of MSI-195 S-Adenosylmethionine (SAMe) was orally administered under fasted conditions, followed by 24 h of blood draws at periodic intervals for the determination of plasma concentration of S-adenosylmethionine in a repeated-measure design. The drug administrations were separated by a wash-out of 7 calendar days.

#### Stage 2 design

In the first 2 periods of this stage of the study, a single 800 mg dose of MSI-195 SAMe and a single 1600 mg dose of commercial comparator (SAM-e Complete™) were orally administered under fasted conditions, in a 2-way crossover design. During the 3rd period, a single 800 mg dose of MSI-195 was administered under fed conditions to all subjects. The drug administrations were separated by a wash-out of 7 calendar days. For each dosing, subjects had periodic blood draws over a 24 h period to determine the plasma concentration of S-adenosylmethionine as a function of time.

#### Study population diagnosis and main criteria of inclusion

Male and female volunteers, non- or ex-smokers, between 21–55 years of age with a body mass index greater than or equal to 18.50 and below 30.00 kg/m2 were included in the study. Subjects were in good health as determined by a medical history, complete physical examination (including vital signs), electrocardiogram (ECG), neurological examination and the usual clinical laboratory tests (general biochemistry, endocrinology, hematology, urinalysis) including negative HIV Ag/Ab Combo, Hepatitis B and Hepatitis C tests (HCV (C)) as well as negative screening of alcohol and drugs of abuse in urine and negative pregnancy test (for female subjects).

### Sample collection – draw timing

In Stage 1 of the study, 18 blood samples were collected as follows: prior to drug administration (t = 0) and 1, 2, 3, 3.5, 4, 4.5, 5, 5.5, 6, 6.5, 7, 8, 9, 10, 11, 12 and 24 h after drug administration. In Stage 2 of the study, for periods under fasting conditions, the 18 blood samples were collected as follows: prior to drug administration (t = 0) and 1, 2, 3, 3.5, 4, 4.5, 5, 6, 7, 8, 9, 10, 11, 12, 14, 16 and 24 h after drug administration, and for the period under fed conditions, the 18 blood samples were collected as follows: prior to drug administration (t = 0) and 1, 2, 3, 4, 5, 6, 7, 8, 9, 10, 11, 12, 14, 16, 18, 21 and 24 h post drug administration.

### Food administration

Food was controlled and standardized throughout the study. The protocol required that subjects start fasting at least 10 h before drug administration for Stage 1 and at least 12 h before drug administration for Stage 2. Fasting continued for 1 h following drug administration in Stage 1, after which a standardized light breakfast was served. A lunch, a supper and a light snack were served at appropriate times thereafter. For Stage 2, all subjects received a standardized low-fat supper before the overnight fasting period. In the first 2 periods, fasting continued for at least 4 h following drug administration, after which a standardized moderate to low fat lunch was served. In the 3rd period, subjects received a standardized high-fat, high calorie meal thirty (30) minutes before drug administration. Further meals were also standardized. In each study period, blood samples were collected over a 24 h period for assessment of SAMe pharmacokinetics. The drug administrations were separated by at least 7 calendar days, in both stages of the study.

### Bioanalytical

#### Handling of samples

Blood samples were collected in pre-cooled K_2_ EDTA Vacutainers. As soon as possible following blood collection, samples were centrifuged at a temperature of 4 °C nominal and at approximately 1500 g for 10 min. A volume of 1.0 mL of plasma was transferred into a polypropylene culture tube containing 40 μL of a solution of 10% H_3_PO_4_, for analyte stability, and was vortexed for at least 5 s. Half of the acidified plasma was then transferred into a second polypropylene tube in order to obtain 2 splits of approximately 0.5 mL of acidified plasma each. Samples were kept in an ice-water bath until frozen in an upright position and retained at a temperature of -80 °C nominal or on dry ice until shipment to the analytical facility. The samples were stored frozen at -80 °C until assayed. The time from blood sample collection to plasma aliquot storage was within 45 min.

#### Method of measurement

Plasma samples were received frozen by the analytical facility (Algorithme Pharma). The experimental samples were assayed for S-adenosylmethionine at the analytical facility using a validated HPLC method with MS/MS detection. The lower limit of quantitation and upper limit of quantitation were 10.0 and 5000.0 ng/mL, respectively.

### Pharmacokinetic assessments

The main absorption and disposition parameters were calculated using a non-compartmental approach with a log-linear terminal phase assumption. The trapezoidal rule was used to estimate area under the curve, and the terminal phase estimation was based on maximizing the coefficient of determination. The pharmacokinetic parameters of this trial were C_max_, (peak drug concentration) T_max_, (time to peak drug concentration),AUC_T_, (Area under the plasma concentration versus time curve from time 0 to 24 h) AUC_∞_, (Area under the plasma concentration versus time curve from time 0 to infinity), K_el_, (terminal elimination rate constant) and T_½_, (elimination half-life).

Statistical analysis of all pharmacokinetic parameters was based on an ANOVA model. Dose proportionality was assessed for Test-1 in Stage 1 of the study.

#### Safety assessments

The safety parameters assessed included the occurrence of adverse events, the measurement of clinical laboratory parameters, vital signs, neurological function tests, physical examination and electrocardiogram (ECG) (See Table 2 for the schedule of assessments). Clinical laboratory parameters (general biochemistry, endocrinology, hematology and urinalysis) were carried out in accordance with Standard Operating Procedures (SOPs) of the licensed laboratory of Laboratoires Bio-Médic. A list of the laboratory variables evaluated at screening and post-study is provided in Table 3. For female subjects, pregnancy tests were also performed prior to the study, during the study and at post-study. Post-study tests were performed after the collection of the last blood sample of the study. Vital signs, ECGs, physical and neurological examinations were performed as described in the protocol. Descriptive statistics was applied.

**Table 2:** Schedule of Assessments for both Stage 1 and 2

	**Pre-Trial**	**Period 1**			**Wash- Out**	**Period 2**			**Wash-Out**	**Period 3**			**End of Study**
Days	-28 to 0	0	1	2	1–7	7	8	9	8–14	14	15	16	16
Informed Consent Form Signed	X												
Admission to Unit		X				X				X			
Medical History	X												
Physical Examination	X												X
Laboratory Tests	X	X				X				X			X
HIV Ag/Ac Combo, HBsAg (B) (Hepatitis B) and anti-HCV (C) Tests	X												
Urine Alcohol and Drugs of Abuse Screening	X	X				X				X			
ECG	X												X
Pregnancy Test	X	X				X				X			X
Vital Signs	X		X				X				X		X
Neurological Function Test	X			X				X				X	
Drug Administration			X				X				X		
Blood Sampling			X	X			X	X			X	X	
Adverse Event Monitoring			X	X	X	X	X	X	X	X	X	X	X

**Table 3:** Laboratory safety assessments evaluated at screening and post-study

**General Biochemistry**	Sodium, potassium, chloride, glucose, urea (BUN), creatinine, total bilirubin, alkaline phosphatase, AST, ALT, albumin
**Endocrinology**	Vitamin B12 and folic acid
**Hematology**	White cell, red cell, hemoglobin, hematocrit, MCV, MCH, MCHC, RDW, platelets, MPV, neutrophil, lymphocyte, monocyte, eosinophil and basophil
**Urinalysis**	Color, appearance, specific gravity, pH, leukocyte, protein, glucose, ketones, bilirubin, blood, nitrite, urobilinogen Microscopic examination only performed if dipstick test is positive for leukocyte, blood, nitrite or protein

### Statistical analysis plan

The natural logarithmic transformation of C_max_, AUC_T_ and AUC_∞_ as well as the rank-transformation of T_max_ was used for all statistical inference. Statistical analyses were generated using SAS® (version 9) using the Mixed procedure.

#### Dose proportionality in stage 1

In Stage 1 of the study a dose proportionality analysis was done on the main PK parameters (C_max_, AUC_T_ and AUC_∞_). These PK parameters were entered in a power model. The power model was defined as:

$$\mathrm{Log e }(\mathrm{Cmax},\mathrm{ AUCT and AUC\infty }) =\mathrm{ \alpha }+\mathrm{ \beta loge}(\mathrm{Dose}) +\upvarepsilon$$

where α is the intercept, β is the slope and ε is the error term. A linear model with log-transformed dose as a continuous effect was fitted. A point estimate and a 90% confidence interval was derived for the slope (β).

## Results

### Demographics

The demographics and baseline clinical characteristics for the volunteers for each stage of the study are listed in Table 4. Stage 1 was a single ascending dose study done in 8 male volunteers with a median age of 37 and median BMI of 24.77. Stage 2 was a 3-period study with a fasted cross-over comparison study of MSI-195 (800 mg) vs. Comparator (SAM-e Complete™, 1600 mg) and a food effect period for MSI-15 (800 mg) in 19 females and 7 males with a median age of 33 and median BMI of 23.39.

**Table 4:** Demographic profile of patients for stages 1 and 2

	**Treatment Groups**	
	**Stage 1** ***N = 8***	**Stage 2** ***N = 26***
**Age (years)**		
Mean ± SD	34 ± 9	35 ± 9
Median	37.0	33.0
Range	21–44	22–55
Groups	n (%)	n (%)
< 18	0 (0%)	0 (0%)
18 – 40	5 (62.5%)	19 (73.1%)
40 – 64	3 (37.5%)	7 (26.9%)
65 – 75	0 (0%)	0 (0%)
> 75	0 (0%)	0 (0%)
**Sex**		
Female	0 (0%)	19 (73.1%)
Male	8 (100)	7 (26.9%)
**Race**		
Asian	1 (12.5%)	0 (0%)
Black	1 (12.5%)	4 (15.4%)
Caucasian	6 (75.0%)	22 (84.6%)
Other	0 (0%)	0 (0%)
**Weight (kg)**		
Mean ± SD	74.6 ± 10.7	64.0 ± 11.6
Median	76.9	61.30
Range	57.7–92.8	48.4–98.0
**Height (cm)**		
Mean ± SD	175.3 ± 10.1	164.1 ± 9.8
Median	175.25	161.25
Range	158.5–189.0	149.0–189.0
**Body Mass Index (kg/m**^**2**^**)**		
Mean ± SD	24.29 ± 2.72	23.63 ± 2.28
Median	24.77	23.39
Range	19.79–27.97	20.15–28.29

### Tolerability and adverse events for stage 1

For Stage 1, all 8 subjects received all 3 doses of MSI-195. MSI-195 was well tolerated at all doses (400, 800 and 1600 mg). The number of subjects experiencing adverse events (AEs) following administration of the 1600 mg dose (*n* = 4; 50.0%) was greater than after administration of the 800 mg (*n* = 0; 0%) or 400 mg (*n* = 2; 25.0%) doses. AEs that occurred at the 1600 mg dose were primarily gastrointestinal (GI) and nervous system disorders, including abdominal pain, upper (*n* = 3), abdominal pain (*n* = 1), flatulence (*n* = 1), eructation (*n* = 1), nausea (*n* = 1) and dizziness (*n* = 2). AEs that occurred at the 400 mg dose level included abdominal pain (*n* = 1), flatulence (*n* = 1), abdominal discomfort (*n* = 1), dizziness (*n* = 1), cough (*n* = 1), upper respiratory infection (*n* = 1), feeling abnormal (*n* = 1), vessel puncture site pain (*n* = 1), blood pressure decreased (*n* = 1) and contusion (*n* = 1). By contrast, no AE was experienced at the 800 mg dose levels of MSI-195.

For Stage 1, all AEs were considered by the investigator to be mild in intensity, with the exception of 1 event of upper abdominal pain, considered moderate in intensity in the 1600 mg dose level. There were no serious AEs reported during this stage of the study, and no subject experienced an AE that resulted in treatment discontinuation or withdrawal. No clinically important changes in laboratory values, vital signs, ECGs, or physical examinations were reported.

### Tolerability and AE’s for stage 2

For Stage 2, the twenty-six subjects were enrolled into a three-treatment, two-sequence, three-period crossover with food effect evaluation study to compare the safety and bioavailability of a single 800 mg dose MSI-195 to that of commercially available SAMe at twice higher dose (1600 mg) level under fasted conditions. Following the completion of the cross-over design, subjects were administered a single 800 mg dose of MSI-195 under fed conditions to evaluate the effect of a high calorie, high fat meal on the bioavailability and pharmacokinetics (PK) of MSI-195. Each subject received MSI-195 (800 mg) once under fed and once under fasting conditions. Twenty-four subjects completed all three treatment periods, which were separated by a 7-day washout.

There were no serious adverse events (SAEs) reported during this stage of the study. Two subjects were withdrawn. One withdrawn subject experienced an AE that resulted in treatment discontinuation (severe vasovagal reaction) in the first (fasted) period (after a 800 mg dose of MSI-195) which resolved approximately 22 h post-dose, and one subject was withdrawn due to pregnancy prior to the second period (and after a dose of MSI-195). No clinically important changes in laboratory values, vital signs, ECGs, physical or neurologic examinations were reported. Twelve subjects experienced an AE in the MSI-195 fasted period, 11 subjects experienced an AE in the commercially available SAMe 1600 mg reference group and 11 subjects experienced an AE in the MSI-195 fed group. AEs that occurred were primarily GI and nervous system disorders. Most AEs were mild in intensity. However, 3 subjects in the MSI-195 fasted period, 2 subjects in the SAMe Comparator (SAM-e Complete™) period and 5 subjects in the MSI-195 fed period reported moderate AEs (abdominal pain, abdominal discomfort, headaches, dizziness). One subject reported severe AE of lower abdominal pain and presyncope in the MSI-195 fasted period. This subject was withdrawn from the study as noted above. AEs considered by the investigator to have a causal relationship with MSI-195 (fasted), SAMe reference (fasted) and MSI-195 (fed) were 72%, 82% and 100% respectively. All GI and nervous system disorders were considered related with the exception of 1 event of nausea and 1 event of dry lip.

Single-dose administration of MSI-195 at oral doses of 800 mg was generally well tolerated in healthy adult volunteers. Adverse events were comparable to those of commercially available SAMe administered at oral doses of 1600 mg. The safety profile of single-dose MSI-195 did not appear to be altered by the consumption of a high fat, high-calorie meal, although the frequency of GI disorders was higher in the MSI-195 fasted period compared to the MSI-195 fed period (26.9% vs 16.7%) and the frequency of nervous system disorders was lower in the MSI-195 fasted period compared to the MSI-195 fed period (11.5% vs. 33.3%).

Most adverse events were considered treatment related. In Stage 1 of the study, 6 subjects experienced adverse events. Only one adverse event of vessel puncture site pain was considered non treatment related. In Stage 2 of the study, 8 subjects experienced treatment-related adverse events in the MSI-195 fasted period and 5 subjects experienced non treatment related adverse events. One event of nausea, dry lip, vessel puncture site reaction, and injury and 2 events of nasal congestion were considered not treatment related. In the MSI-195 fed period, all adverse events were considered treatment-related. See Table 5 for a summary of adverse events.

**Table 5:** Reported incidence of adverse events, by treatment group, by at Least 1 Subject Treated with MSI-195 or SAM-e Complete™ for stages 1 and 2

	**Stage 1**			**Stage 2**		
**Body System/ Adverse Event**	MSI-195 400 mg (*N* = 8)	MSI-195 800 mg (*N* = 8)	MSI-195 1600 mg (*N* = 8)	MSI-195 800 mg (*N* = 26) Fasted	SAM-e Complete™ 1600 mg (*N* = 24)	MSI-195 800 mg (*N* = 24) Fed
Subjects with at least one AE [n(%)]	2 ( 25.0)	0	4 ( 50.0)	12 ( 46.2)	11 ( 45.8)	11 ( 45.8)
**GASTROINTESTINAL DISORDERS [n(%)]**	2 ( 25.0)	0	4 ( 50.0)	7 ( 26.9)	5 ( 20.8)	4 ( 16.7)
Abdominal Pain Upper [n(%)]	0	0	3 ( 37.5)	0	0	0
Abdominal Pain [n(%)]	1 ( 12.5)	0	1 ( 12.5)	2 ( 7.7)	1 ( 4.2)	2 ( 8.3)
Abdominal Pain, Lower [n (%)]				1 ( 3.8)	2 ( 8.3)	0
Flatulence [n(%)]	1 ( 12.5)	0	1 ( 12.5)	0	0	0
Abdominal Discomfort [n(%)]	1 ( 12.5)	0	0	1 (3.8)	1 (4.2)	1 (4.2)
Abnormal Faeces [n(%)]	0	0	0	0	1 ( 4.2)	0
Dyspepsia [n(%)]	0	0	0	1 ( 3.8)	0	0
Lip Dry [n(%)]	0	0	0	1 ( 3.8)	0	0
Vomiting [n(%)]	0	0	0	1 ( 3.8)	0	0
Eructation [n(%)]	0	0	1 ( 12.5)	0	0	0
Nausea [n(%)]	0	0	1 ( 12.5)	4 ( 15.4)	2 ( 8.3)	1 ( 4.2)
**NERVOUS SYSTEM DISORDERS [n(%)]**	1 ( 12.5)	0	2 ( 25.0)	3 ( 11.5)	4 ( 16.7)	8 ( 33.3)
Dizziness [n(%)]	1 ( 12.5)	0	2 ( 25.0)	1 ( 3.8)	1 ( 4.2)	0
Somnolence [n(%)]	0	0	0	2 ( 7.7)	2 ( 8.3)	6 ( 25.0)
Headache [n(%)]	0	0	0	1 ( 3.8)	2 ( 8.3)	4 ( 16.7)
Dysgeusia [n(%)]	0	0	0	0	1 ( 4.2)	1 ( 4.2)
Presyncope [n(%)]	0	0	0	1 (3.8)	0	0

### Pharmacokinetic results-stage 1

**Fig. 1:**
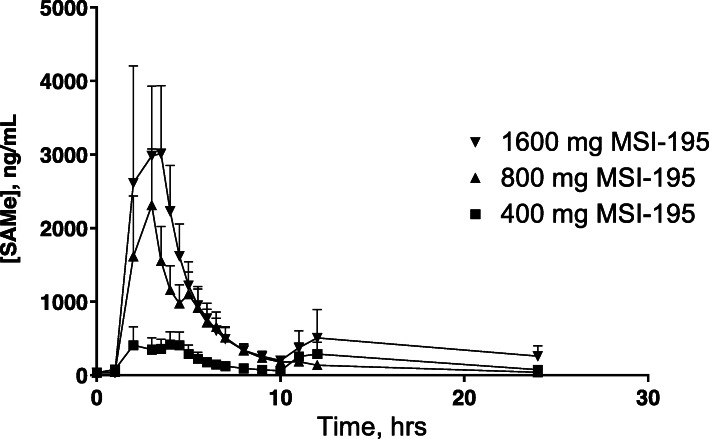
Mean S-adenosylmethionine (SAMe) plasma concentration vs time profile following oral administration of 400 mg (1 tablet), 800 mg (2 tablets) or 1600 mg (4 tablets) of the SAMe formulation MSI-195 to 8 fasted healthy male volunteers in Stage 1. Error bars are standard error of the mean

Following administration of MSI-195 by oral administration at doses of 400 mg, 800 mg or 1600 mg to healthy volunteers approximately linear pharmacokinetics was observed based on mean plasma C_max_, AUC_T_ and AUC_0-∞_ (Table 6). The average pharmacokinetic curves for each period are shown in Fig. 1. The increase in AUC is close to, but slightly less than, dose-proportional. The variability in parameters within groups may be responsible for this divergence from the expected relationship. The AUC_T_ were 4643.9 ng*h/ml, 9360.6 ng*h/ml and 16,511 ng*h/ml for 400 mg, 800 mg and 1600 mg, respectively. The elimination half-life showed some variability, potentially related to dose escalation, providing values 16.01 h, 8.87 h and 7.77 h for 400 mg, 800 mg and 1600 mg, respectively.

**Table 6:** Summary of pharmacokinetic parameters for S-Adenosylmethionine oral dosage forms for single dosing in Stages 1 and 2. Values are presented as the mean with the standard deviation (SD)

	Treatment					
	MSI-195 400 mg		MSI-195 800 mg		MSI-195 1600 mg	
Study Stage 1						
Variable	Mean	SD	Mean	SD	Mean	SD
C_max_, ng/mL^a^	1045.4	601.8	3255.5	2347.1	4808.8	4028.2
T_max_, h	7.75	9.12	3.00	2.90	3.25	7.81
AUC_T_, ng h/mL	4643.9	2519.2	9360.6	5338.9	16,511.0	10,736.0
AUC_∞_, ng h/mL^b^	5322.1	956.1	10,633.2	5147.3	16,454.2	10,675.7
K_el_, h^−1^	0.0465	0.01	0.0886	0.04	0.1056	0.05
T_1/2_, h	16.01	4.90	8.87	3.08	7.77	3.26
Study Stage 2						
	MSI-195 800 mg Fasted		MSI-195 800 mg Fed		SAMe 1600 mg Fasted	
Variable	Mean	SD	Mean	SD	Mean	SD
C_max_, ng/mL	3280.8	2383.8	1461.0	1153.7	1984.3	1430.0
T_max_, h	4.50	1.72	13.00	6.03	6.00	1.23
AUC_T_, ng h/mL	9434.4	5196.1	5224.7	3094.6	6643.8	3326.4
AUC_∞_, ng h/mL	10,025.5	4799.5	7445.4	2688.2	7144.4	3214.2
K_el_, h^−1^	0.1007	0.08	0.2322	0.07	0.0926	0.04
T_1/2_, h	11.47	7.66	3.30	1.37	9.27	4.59

^a^For C_max_, the arithmetic mean is reported

^b^For elimination parameters, *n* = 4 for the 400 mg dose, *n* = 7 for the 800 mg dose and *n* = 6 for the 1600 mg dose

Large variability in the T_max_ was observed between the three dose levels and variability within groups was also high. Upon examination of the individual data sets it was observed that this variability may be related to delayed gastric emptying in specific subjects. Based on this data, a more stringent fasting schedule (from fasting for 1 h before dosing to 4 h before dosing) was used in Stage 2 of this study in an attempt to reduce the variability.

The low number of subjects (*N* = 8), coupled with the high variability of the data limits the ability to discern statistically significant differences amongst the treatment periods. An ordinary one-way ANOVA analysis of the three groups indicated that the area under the curve for the three groups were significantly different however, with a P value of 0.0107 and F score of 5.681. Multiple comparison analysis between groups (Tukey’s multiple comparisons test) showed no significant difference between 400 and 800 mg or between 800 and 1600 mg (adjusted P values of 0.3952 and 0.1325 respectively) but did show a significant difference between the 400 mg and 1600 mg periods with a P value of 0.0082.

### Pharmacokinetic results-stage 2

**Fig. 2:**
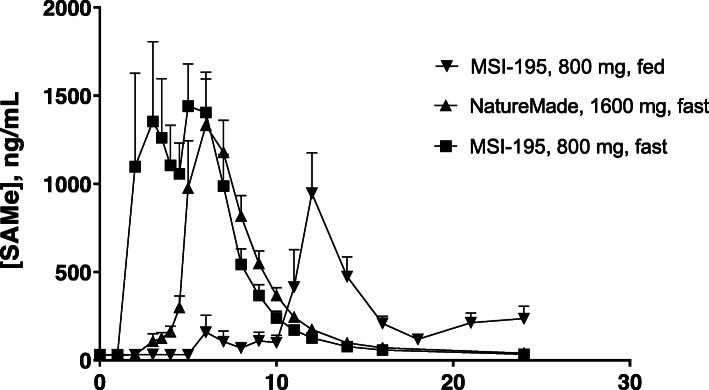
Mean S-adenosylmethionine (SAMe) plasma concentration vs time profile following oral administration of 800 mg (2 tablets) of the SAMe formulation MSI-195 under fasted or fed conditions or 1600 mg (4 tablets) of SAM-e Complete™ under fasted conditions to 24 healthy male and female volunteers in Stage 2. Error bars are standard error of the mean

The average pharmacokinetic profile for each of the 3 periods in Stage 2 is shown in Fig. 2. In Stage 2 of this trial, comparing fed and fasted in addition to comparison with the commercial SAMe formulation, the mean MSI-195 T_max_ was 4.50 h in the fasted state but was significantly delayed to 13.00 h in the fed state (Table 6). Administration of 800 mg MSI-195 resulted in an average C_max_ of 3280.8 ng/mL under fasted conditions and 1461.0 ng/mL when fed. The AUC_T_ was 9434.4 ng/ml and 5224.7 ng/ml under fasted and fed conditions, respectively. After reaching C_max_, plasma levels of SAMe decrease relatively slowly, followed by a terminal-plasma elimination half-life (T½) of 11.8 h under fasted conditions and 3.30 h under fed conditions. The delayed T_max_ resulted in sub-optimal data for the calculation of the full elimination profile. This may have in turn affected the accuracy of the calculated PK parameters under fed conditions. PK parameters were generally quite variable, but the variability was comparable to the commercial formulation (SAM-e Complete™) of SAMe used as a comparator. On a per mg basis, MSI-195 under fasted conditions gave approximately threefold greater exposure than commercial SAMe as judged by C_max_ (3.31-fold higher), AUC_T_ (2.84-fold higher and AUC_∞_ (2.80-fold higher). The T_max_ for MSI-195 was somewhat shorter than seen with the commercial material.

Stage 2 of the study included 17 females and 7 males in the analysis. The descriptive gender analysis of the pharmacokinetic parameters (Table 7) indicated that gender appears to influence the mean C_max_ value of both MSI-195 and SAM-e Complete™ formulation and also had an impact on the AUC values. The bioavailability of MSI-195 was higher in males than in females as shown by a higher C_max_ and AUC. The reverse was observed with SAM-e Complete™ where the bioavailability was greater in females (higher C_max_ and AUC) than in males. The median time to reach the maximum plasma concentrations appeared to be slightly slower in female subjects, i.e. 1 h difference for both products. Finally, the half-life was similar in both male and female subjects.

**Table 7:** Gender Effect Evaluation of S-Adenosylmethionine, Stage 2

PARAMETER	MSI-195				SAM-e Complete™	
	**800 mg**				**1600 mg**	
	**Fasting**		**Fed**		**Fasting**	
	**Male**, (*n* = 7)	**Female**, (*n* = 17)	**Male**, (*n* = 7)	**Female**, (*n* = 17)	**Male**, (*n* = 7)	**Female**, (*n* = 17)
Cmax (ng/mL)	3832.0	3053.9	1144.4	1591.3	1205.8	2304.9
Tmax (hours) *	3.50	4.50	12.00	14.00	5.00	6.00
AUC_t_ (ng·h/mL)	8693.5	8743.1	4599.2	5482.3	5098.7	7182.7

The gender stratified descriptive analysis of the pharmacokinetic parameters showed that the female subjects had a higher C_max_ and AUC in fed conditions than males, and males showed higher C_max_ and AUC in fasting conditions than their female counterparts. Overall, the effect of food on the PK profile of MSI-195 was greater in males in terms of maximal concentrations and extent of absorption; their mean C_max_ tripled and their mean AUC doubled in fasting conditions compared to fed. In both genders, T_max_ was similar in both conditions.

Statistical analysis of MSI-195 at 800 mg vs. SAM-e CompleteTM at 1600 mg, both under fasted conditions showed that the AUC_t_ was significantly different as indicated by a paired t test of all 24 completing subjects with a P value of 0.0180. Note that the data were not dose adjusted. The 800 mg of the MSI-195 was statistically significantly higher in AUC_t_ compared with the 1600 mg of SAM-e Complete™.

## Discussion

The results of this study show that the relative bioavailability of the MSI-195 formulation of S-adenosylmethionine is 2.8 times higher than the comparator SAM-e Complete™ product. In the cross-over, fasted portion of Stage 2, the AUC_T_ for MSI-195 with an 800 mg dose was 9,434.5 ng hr/mL or 11.80 ng hr/mL on a per mg basis. For the SAM-e Complete™ comparator, the AUC_T_ with a 1600 mg dose was 6,643.8 ng hr/mL or 4.15 ng hr/mL on a per mg basis. That data indicates a 2.8 fold higher relative bioavailability for the MSI-195 formulation.

In Stage 1, oral administration at doses of 400 mg, 800 mg or 1600 mg to healthy volunteers showed approximately linear MSI-195 SAMe pharmacokinetics based on mean plasma C_max_, AUC_T_ and AUC_(0-∞)_. The AUC_T_ were 4643.9 ng*h/ml, 9360.6 ng*h/ml and 16,511.0 ng*h/ml for 400 mg, 800 mg and 1600 mg, respectively. The elimination half-life showed some variability, potentially related to dose escalation, providing values 16.01 h, 8.87 h and 7.77 h for 400 mg, 800 mg and 1600 mg, respectively. Repeat dose studies of MSI-195 have not been carried out at this time. Studies with other formulations of SAMe suggested that repeated dosing has no impact on SAMe exposure over time [12].

### Bioavailability comparison to literature

Studies examining SAMe human pharmacokinetics have been published by several groups [12, 16–18]. While different methodologies used in these different studies are bound to lead to some anomalies, comparisons may be useful. The relatively high exposure on a per mg basis in comparing data with MSI-195 is immediately of note (Table 8). As discussed, MSI-195 provides approximately 2.8-fold greater exposure than commercial SAMe (SAM-e Complete™) and a similar increase in exposure is seen relative to the data published by Yang et al. [12]. The study by Loehrer et al. achieved much lower exposure [17]. Intravenous and intramuscular administration of SAMe in these literature studies obviously achieved far higher plasma concentrations and, while the results from different studies are variable, some concordance is seen (See Table 8).

**Table 8:** Dose corrected comparison of SAMe PK data across studies

	Reference	Route	Dose	Parameters			Parameters Corrected for Dose	
				C_max_	AUC^a^	T_1/2_	C_max_	AUC_T_
				ng/ml	ng/ml*h	h	(ng/ml)/mg	(ng/ml*h)/mg
MSI Study	Stage 1:							
	MSI-195	Oral	400	1045.4	4643.9	16.0	2.6	11.6
	MSI-195	Oral	800	3255.5	9360.6	8.9	4.1	11.7
	MSI-195	Oral	1600	4808.8	16,511.0	7.8	3.0	10.3
	Stage 2:							
	MSI-195 Fast	Oral	800	3280.8	9434.4	11.5	4.1	11.8
	MSI-195 Fed	Oral	800	1461.0	5224.7	33	1.8	6.5
	SAMe (NM)	Oral	1600	1984.3	6643.8	9.3	1.2	4.2
Literature Studies	Loehrer et al. [17]	Oral	400	144	489	1.7	0.4	1.2
	Yang et al. [12]	Oral	1000	974	4748	6.2	1.0	4.7
	Yang et al. [12]	IV	1000	67,600	162,000	4.1	67.6	162.0
	Guilidori et al. [16]	IV	100	8320	7833	1.35	83.2	78.3
	Guilidori et al. [16]	IV	500	31,300	42,500	1.68	62.6	85.0
	Stramentinoli et al. [18]	IM	35	-	4300	1.35	-	122.9
	Stramentinoli et al. [18]	IV	35	-	4000	1.35	-	114.3

The sponsor has not carried out clinical studies to specifically estimate the oral bioavailability of MSI-195. However, this can be estimated from literature that assessed intravenous administration of SAMe. By using the mean AUC from the four intravenous studies detailed in Table 8, the oral bioavailability of MSI-195 (based on 1600 mg in Stage 1) is approximately 9%. Clearly this is a very approximate assessment of the absolute oral bioavailability. Comparative literature on absolute bioavailability for other formulations of S-adenosylmethionine is extremely limited. Yang and co-workers reported a oral to iv ratio of 2.6% and 2.14% for single and multiple oral dosing respectively [12].

### Food effect

The T_max_ for enteric coated oral dosage forms is highly dependant on gastric emptying which is a stochastic event dependent on the density, size and shape of the dosage form, as well as the age, gender and fed state of the subject [19–21]. The pharmacokinetics of MSI-195 with a high fat meal, including the delayed and variable T_max_, are fully consistent with the well-studied pattern of gastric emptying of other enteric-coated tablets, for example, as reported in the FDA labeling for Depakote [22], EC-Naprosyn [23] and Voltaren [24]. This variable and extended delay with enteric-coated tablets given with food is typically attributed to variable gastric emptying of non-disintegrating dosage forms in the fed state. This variability in transit from the stomach to the GI tract is also evident in the fasted periods of this study. The T_max_ for SAM-e dosage forms, which are all approximately 1 g, enteric coated tablets is highly variable. This peak timing variability between subjects is the primary cause of the appearance of the “double peaks” observed on the average PK curves in Figs. 1 and 2.

### Gender effect

Stage 2 of the study included 17 females and 7 males. Judging by the gender-specific pharmacokinetic parameters, it appears that gender may influence the mean C_max_ value of both MSI-195 and SAM-e Complete™ formulation and also impact on the AUC values. The bioavailability of MSI-195 was higher in males than in females as shown by a higher C_max_ and AUC. The reverse was observed with SAM-e Complete™ where the bioavailability was greater in females (higher C_max_ and AUC than in males). The median time to reach the maximum plasma concentrations appeared to be slightly slower in female subjects, i.e. 1 h difference for both products. Finally, the half-life was similar in both male and female subjects.

Given the lack of consistent directionality to the gender effects observed in this study, the low number of subjects and the high variability of the pharmacokinetic properties for SAM-e formulations generally, more study would be required to draw reliable conclusions regarding gender effects for the studied formulations of SAM-e.

### Safety

Single-dose administration of MSI-195 at oral doses of 400, 800 and 1600 mg was well tolerated in healthy adult volunteers. Adverse events were comparable to those of commercially available SAMe administered at oral doses of 1600 mg. The safety profile of a single-dose MSI-195 did not appear to be altered by the consumption of a high-fat, high-calorie meal, although the frequency of GI disorders was higher in the MSI-195 fasted period compared to the MSI-195 fed period (26.9% vs 16.7%) and the frequency of nervous system disorders was lower in the MSI-195 fasted period compared to the MSI-195 fed period (11.5% vs. 33.3%). The most frequently observed adverse events experienced by subjects and judged to be possibly related to MSI-195 were gastrointestinal in nature. (see Table 5).

The severity of adverse events ranged from mild to severe during this study. Two (2) severe adverse events (abdominal pain lower and syncope) were observed on the same subject following the 800 mg MSI-195 administration under fasting conditions. This subject was withdrawn from the study for safety reasons (Investigator's decision) following the vasovagal reaction.

No serious adverse events or deaths were reported during this study. No clinically significant effects on laboratory evaluations, ECGs, physical examinations or neurological function tests, were noted during this study. Following the single 400 mg oral dose administration of MSI-195 one (1) subject experienced a clinically significant decrease in blood pressure at 2.50 h post-dose; the value came back within normal range after a few minutes.

In Stage 1 of the study (single ascending doses of MSI-195), the highest rate of subjects reporting adverse events was observed with the highest dose of MSI-195 (1600 mg), followed by the lowest dose (400 mg). However, as no adverse event was reported with the intermediate dose (800 mg). No clear pattern of increasing frequency of adverse events with increasing doses was observed during this study.

In the crossover periods of Stage 2, between MSI-195 800 mg and SAM-e Complete™ 1600 mg under fasting conditions, slightly more adverse events were reported with the administration of MSI-195 compared to the administration of SAM-e Complete™. However, this difference was mainly due to the multiple adverse events experienced by one subject (the subject with the vasovagal reaction mentioned earlier) following the administration of MSI-195. Thus, the safety profile of both products in fasting conditions appears more or less similar at the doses administered during this study.

In the food-effect evaluation on MSI-195 (in Stage 2), it was observed that fewer adverse events were reported when MSI-195 800 mg was administered under fed conditions compared to when it was administered under fasting conditions. Here again the same conclusion can be made as one subject (the subject with the vasovagal reaction mentioned earlier) experienced multiple adverse events when administered MSI-195 under fasting conditions. The observations made in this study lead to the conclusion that MSI-195 could be administered to patients in either fed or fasted conditions with a comparable safety outcome.

### Bioavailability enhancement

The mechanism of bioavailability enhancement for MSI-195 is directly related to the presence of propyl gallate. The molecular mechanism by which propyl gallate confers the higher bioavailability for SAMe disulfate tosylate is not known but may involve facilitating paracellular transport of SAMe [25]. MSI has observed in separate studies in beagles (data not shown) that propyl gallate needs to be co-formulated with SAMe in order to enhance systemic bioavailability. Given the basis of this selectivity for SAMe disulfate tosylate, concomitantly administered agents are not anticipated to exhibit increased absorption due to the presence of propyl gallate.

### Study limitations

The sample sizes for both stages of this study were small, which is a limitation.

## Conclusions

MSI-195 was well tolerated at single doses both with and without food, showed close to linear dose-dependant pharmacokinetics and demonstrated significantly higher relative bioavailability relative to the SAM-e Complete™ commercial comparator in a cross-over study as well higher relative bioavailability when compared to literature data.

## Data Availability

The data generated and analyzed within this study is available from the corresponding author on request.

## Abbreviations

AE: Adverse event
AUC∞: Area under the plasma concentration versus time curve from time zero to infinity
AUCT: Area under the plasma concentration versus time curve from time zero to 24 h
Cmax: Peak drug concentration
ECG: Electrocardiogram
GI: Gastrointestinal
im: Intramuscular
iv: Intravenous
Kel: Terminal elimination rate constant
PK: Pharmacokinetics
SAE: Serious adverse event
SAMe: S-adenosylmethionine
SD: Standard deviation
SOP: Standard operating procedure
T1/2: Elimination half life
Tmax: Time to peak drug concentration
